# Right Subclavian Artery With Kommerell's Diverticulum: A Rare Cause of Dysphagia

**DOI:** 10.7759/cureus.35812

**Published:** 2023-03-06

**Authors:** Franca Erhiawarie, Ogochukwu E Chioma, Omar Rahim, Sahar Ansar, Uzochukwu Adabanya, Adekunle E Omole, Ayoola Awosika

**Affiliations:** 1 Internal Medicine, University of Benin, Benin, NGA; 2 Surgery, Federal Medical Center Keffi, Nasarawa State, NGA; 3 Internal Medicine, Naseer Teaching Hospital, Peshawar, PAK; 4 Internal Medicine, Islamic International Medical College, Rawalpindi, PAK; 5 Community Medicine, Mercer University School of Medicine, Columbus, USA; 6 Anatomical Sciences, American University of Antigua, College of Medicine, Saint John, ATG; 7 College of Medicine, University of Illinois, Chicago, USA; 8 College of Health Sciences and Professions, Ohio University, Athens, USA

**Keywords:** esophageal stenosis, dysphagia lusoria, abnormal branching of the subclavian artery, aortic arch variant, kommerell's diverticulum

## Abstract

Kommerell's diverticulum is an embryologic developmental anomaly of the aortic arch wherein a diverticulum arises from either the left or the right aortic arch. It results due to the persistence of the remnant of the fourth dorsal aortic arch. We present a case of a 66-year-old female presenting with complaints of throat pain and difficulty swallowing. A computed tomography (CT) scan of the neck with contrast revealed an incidental finding of an aberrant right subclavian artery with associated diverticula of Kommerell, measuring up to 1 cm, causing a mass effect on the esophagus and posterior trachea. A diagnosis of dysphagia lusoria was established, and an upper gastrointestinal (GI) series revealed narrowing of the esophagus from posterior extrinsic compression. The patient was discharged home for nutrition optimization with a percutaneous endoscopic gastrostomy (PEG) tube due to significant weight loss from the inability to swallow before proceeding with surgery to repair the aberrant right subclavian artery.

## Introduction

Kommerell's diverticulum is an embryologic developmental anomaly of the aortic arch wherein a diverticulum arises from either the left or the right aortic arch. Burckhard Friedrich Kommerell initially described it in 1936 as a pulsatile mass compressing the esophagus from its posterior aspect [1]. It results due to the persistence of remnant of the fourth dorsal of the brachiocephalic trunk. The right subclavian artery arises as a branch of the aortic arch and courses from the proximal descending aorta to the right arm, passing behind the esophagus and leading to its compression. Aortic arch developmental anomalies, in general, are estimated to be at 1-3 % in newborns born with congenital heart disease [2]. The prevalence of aberrant right and left subclavian arteries constitutes 0.7-2% and 0.4% of the population [3]. The histology of the specimen shows the presence of medial cystic necrosis in the diverticulum wall [4]. Here, we discuss a case where the diverticulum causes pressure on the trachea and esophagus, causing decreased nutritional intake and compressive atelectasis in the lungs mimicking mass lesions.

## Case presentation

**Figure 1:**
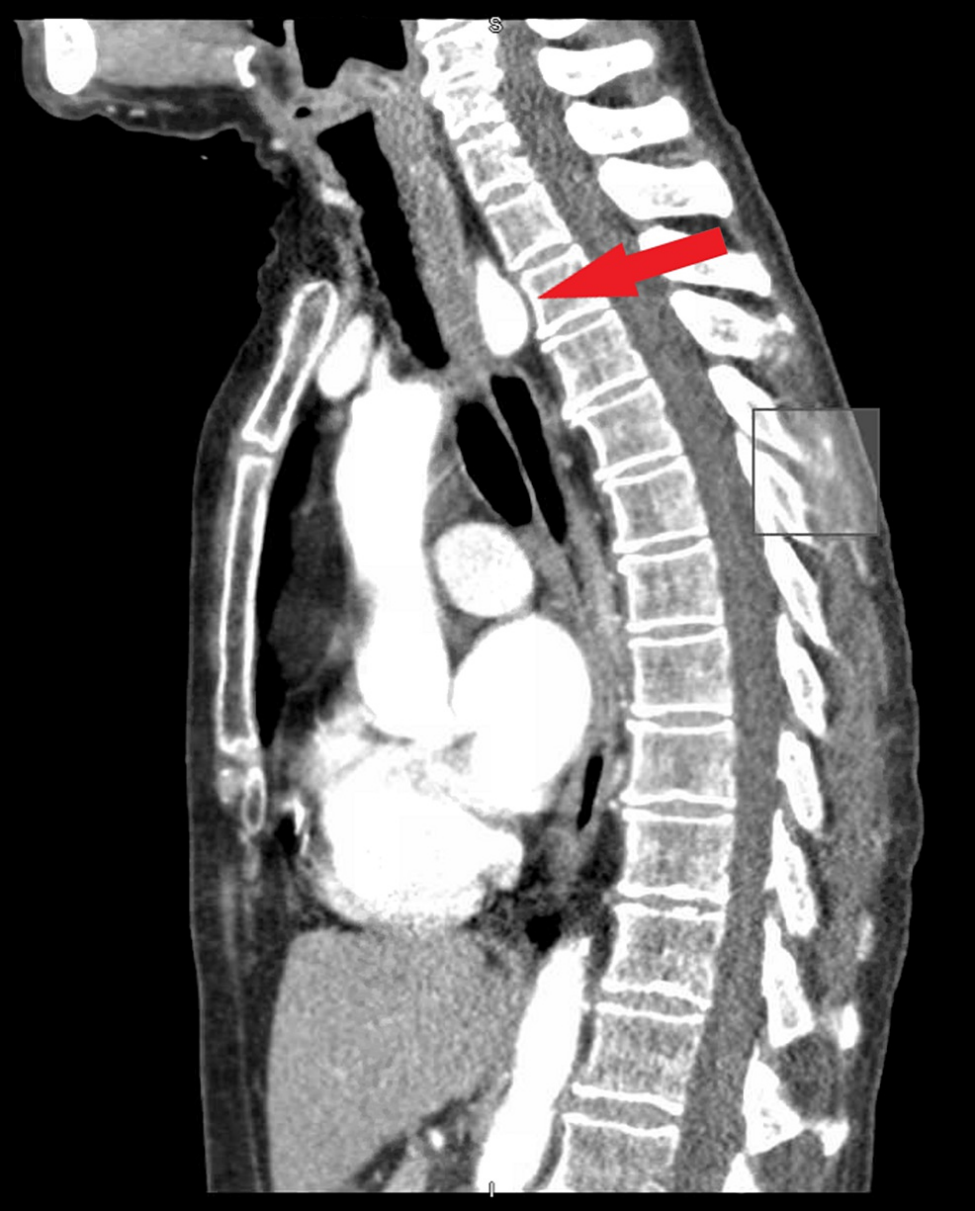
CT neck showing Kommerell diverticula

A 66-year-old female with a past medical history significant for extensive smoking, depression, and fibromyalgia presented to the emergency department with complaints of throat pain, dysphagia, and inability to eat, ongoing for six months. She reported over 30 pounds of unintentional weight loss. Additionally, she found her voice getting coarse over time. On admission, the patient was vitally stable, normothermic, and sating well on room air. The physical examination showed bilateral wheezing with decreased air entry to the right lung. Labs were unremarkable. Computed tomography (CT) scan of the neck revealed an aberrant right subclavian artery with associated diverticula of Kommerell, measuring up to 1 cm with an associated mass effect on the esophagus and posterior trachea (Figure 1).

**Figure 2:**
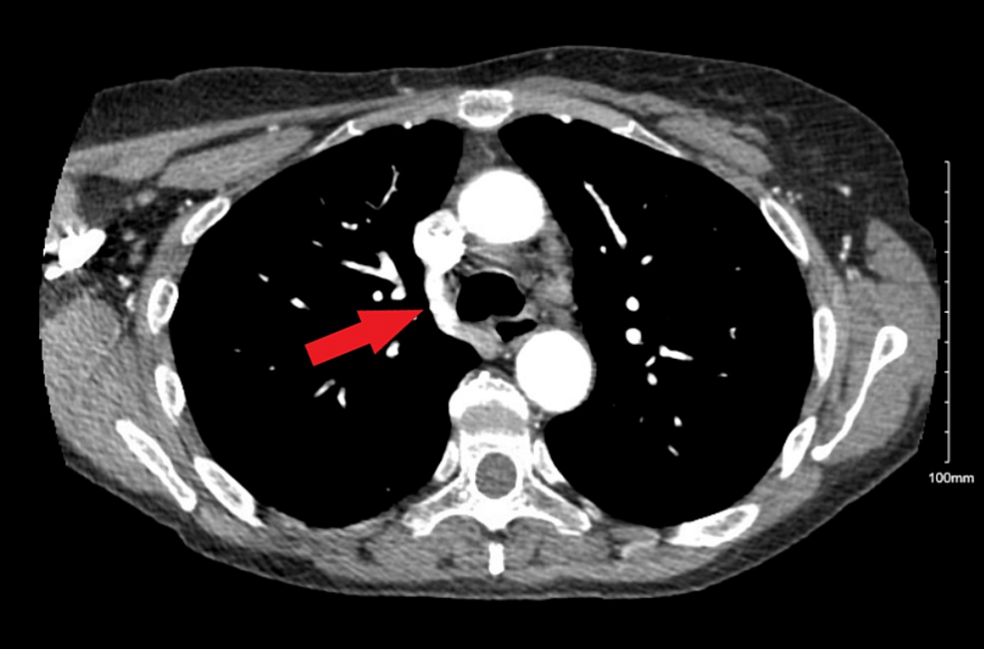
CT angiogram showing an aberrant right subclavian artery

Atherosclerotic calcifications were seen throughout the visualized vasculature on a CT angiogram of the chest, which confirmed the vascular ring (Figure 2).

**Figure 3:**
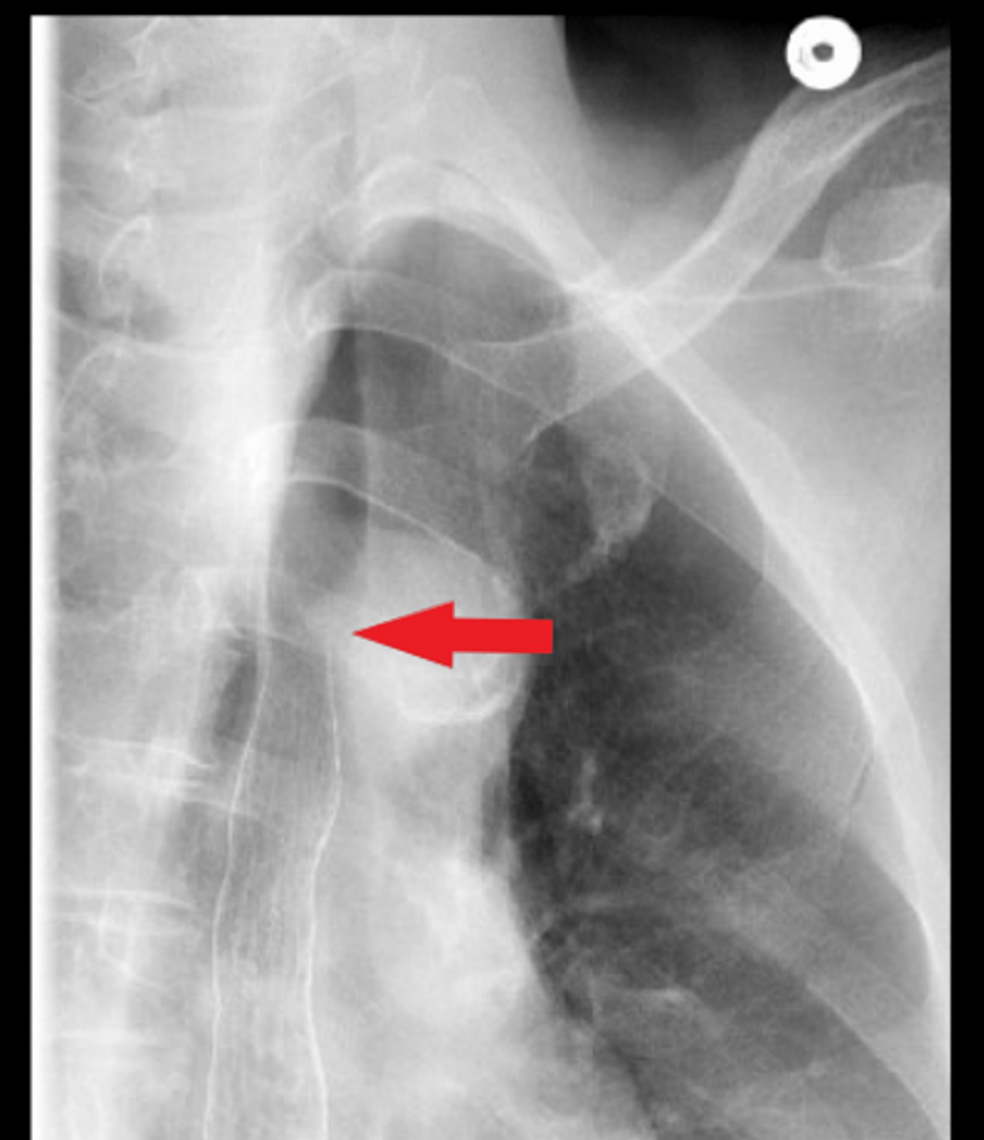
Esophagogram showing esophageal compression

Dysphagia lusoria was considered the diagnosis. An upper gastrointestinal (GI) series revealed a narrowing of the esophagus from posterior extrinsic compression (Figure 3).

Endoscopy was performed, which showed third-degree esophageal contraction and duodenitis. Vascular surgery was consulted, who recommended optimization of nutrition with a percutaneous endoscopic gastrostomy (PEG) tube due to significant weight loss from the inability to swallow before proceeding with surgery to repair the aberrant right subclavian artery. The patient was discharged home with a PEG tube and after six months, the patient underwent a hybrid repair using an aortic endograft and extra-anatomic bypass due to significant comorbidity and avoiding the long-term complications with open repair.

## Discussion

Kommerell's diverticulum is usually reported in neonatal and pediatric age groups. It is a rare congenital anomaly that is caused by the absence of involution of the primitive fourth distal aortic arch where an aberrant right subclavian artery arises from the proximal descending aorta, causing compression of the esophagus and trachea. Our case is highly unusual, given the presenting age of the patient, which may be attributed to atherosclerotic calcification preserving the structure of the artery. It is prudent to identify this diverticulum early, as it may lead to the formation of an aortic aneurysm and may rupture, causing aortic dissection [5]. Kommerell's diverticulum is also known as "lusoria diverticulum," "remnant diverticulum," or "lusoria root" [1]. There are three different subtypes of aortic arch diverticulum: a left-sided aortic arch with an aberrant right subclavian artery, a right-sided aortic arch with an aberrant left subclavian artery, and a diverticulum at the aortic-ductal junction [6]. Given its presentation is similar to oropharyngeal malignancy, additional workup should be done with an esophagogram and endoscopy to rule out serious etiologies. Kommerell's diverticulum is a very rare abnormality, present in only 0.36% of the patients with dysphagia [7]. It can be asymptomatic or present with symptoms of compression of mediastinal structures, including the esophagus and trachea. Children usually present with airway symptoms while adults most commonly present with dysphagia and chest discomfort [4].

Kommerell's disease is a congenital abnormality and usually presents early but our case is unique in the fact that it was detected incidentally on imaging in a 66-year-old patient. A thorough understanding of the underlying anatomy is important, as it is frequently associated with other congenital arterial abnormalities like a right-sided aortic arch or a duplicated arch. The course of the aberrant right subclavian artery is usually posterior to the esophagus but it can be retro-tracheal or sometimes can be anterior to the trachea. This leads to dysphagia or dyspnea because of the compromise of the neighboring structures. The symptoms may manifest late in life as the development of atherosclerotic rigidity and tortuosity, especially if the aberrant right subclavian artery originates from a diverticulum [8]. This is what was seen in our patient, where she was found to be having atherosclerotic changes on the angiogram. There is no established management guideline due to the rarity of the condition. Some options for surgical repair include aortic replacement, thoracic endovascular stent-graft replacement (TEVAR), and extra-anatomic bypass of the aberrant subclavian artery [9]. Surgical repair is considered when the diameter of the diverticulum orifice exceeds 30 mm [10]. A left subclavian carotid bypass is recommended before the surgical repair to avoid a subclavian steal syndrome [11].

## Conclusions

Kommerell's diverticulum is a rare cause of dysphagia in patients from the external compression of the esophagus by abnormal development of the aortic arch. This can lead to pressure symptoms on the trachea or esophagus leading to hoarseness or dysphagia. Surgical repair is considered when the diameter of the diverticulum orifice exceeds 30 mm or when the patient is having severe symptoms of dysphagia or hoarseness.
